# FERONIA functions through Target of Rapamycin (TOR) to negatively regulate autophagy

**DOI:** 10.3389/fpls.2022.961096

**Published:** 2022-08-23

**Authors:** Ping Wang, Natalie M. Clark, Trevor M. Nolan, Gaoyuan Song, Olivia G. Whitham, Ching-Yi Liao, Christian Montes-Serey, Diane C. Bassham, Justin W. Walley, Yanhai Yin, Hongqing Guo

**Affiliations:** ^1^Department of Genetics, Development and Cell Biology, Iowa State University, Ames, IA, United States; ^2^Department of Plant Pathology and Microbiology, Iowa State University, Ames, IA, United States; ^3^Plant Sciences Institute, Iowa State University, Ames, IA, United States

**Keywords:** Autophagy, AZD8055, FERONIA, S6K1 phosphorylation, TOR

## Abstract

FERONIA (FER) receptor kinase plays versatile roles in plant growth and development, biotic and abiotic stress responses, and reproduction. Autophagy is a conserved cellular recycling process that is critical for balancing plant growth and stress responses. Target of Rapamycin (TOR) has been shown to be a master regulator of autophagy. Our previous multi-omics analysis with loss-of-function *fer-4* mutant implicated that FER functions in the autophagy pathway. We further demonstrated here that the *fer-4* mutant displayed constitutive autophagy, and FER is required for TOR kinase activity measured by S6K1 phosphorylation and by root growth inhibition assay to TOR kinase inhibitor AZD8055. Taken together, our study provides a previously unknown mechanism by which FER functions through TOR to negatively regulate autophagy.

## Introduction

FERONIA (FER) is a receptor-like kinase (RLK) and belongs to the *Catharantus roseus* RLK1-like (CrRLK1L) subfamily. FER functions together with co-receptors LLGs/LRE, cell wall components, such as LRX proteins and pectin, and ligands such as RALFs to regulate many downstream signaling components to play essential roles in plant growth and development, reproduction and stress responses (Boisson-Dernier et al., 2011; Lindner et al., 2012; Haruta et al., 2014; Li et al., 2016; Franck et al., 2018; Zhao et al., 2018; Duan et al., 2020; Solis-Miranda and Quinto, 2021; Lin et al., 2022; Xie et al., 2022). FER was first identified for its crucial role in female reproduction, as loss-of-function *fer* mutants displays reduced fertility due to pollen tube overgrowth (Escobar-Restrepo et al., 2007). FER was also required for optimal vegetative growth (Guo et al., 2009) and root hair development (Duan et al., 2010). Subsequent studies revealed more functions of FER, including plant hormone Abscisic Acid (ABA) signaling (Yu et al., 2012), cold and heat stress responses (Chen et al., 2016), salt stress response (Feng et al., 2018; Zhao et al., 2018; Gigli-Bisceglia and Testerink, 2021), photooxidative stress (Shin et al., 2021), mechano-sensing (Shih et al., 2014; Tang et al., 2022), thigmomorphogenesis (Darwish et al., 2022) and immune responses to bacterial pathogens (Keinath et al., 2010; Stegmann et al., 2017; Guo et al., 2018), fungal pathogens (Kessler et al., 2010; Masachis et al., 2016) and nematodes (Zhang et al., 2020). Recent study showed that FER-mediated ROS production regulates levels of beneficial pseudomonads in the rhizosphere microbiome, independent of FER’s immune function (Song et al., 2021). Our most recent study also revealed novel functions of FER in the negative regulation of Endoplasmic Reticulum (ER) body formation and glucosinolate biosynthesis (Wang et al., 2022).

Autophagy, meaning “self-eating,” is a conserved cellular recycling process that employs specialized vesicles to encapsulate and deliver cytoplasmic material to the vacuole for degradation. Cargoes including single macromolecules, large macromolecular complexes such as ribosomes and proteasomes, protein aggregates, damaged or whole organelles, and even invading pathogens can be eliminated through autophagy in bulk or selectively (Liu and Bassham, 2012; Marshall and Vierstra, 2018). This autophagic process is critical for removing non-functional molecules within the cell and replenishing the nutrient sources for new growth, and it is also important for regulating specific biological processes or signaling pathways by selectively degrading specific cargoes (Zhou et al., 2014; Nolan et al., 2017; Zhuang et al., 2018; Xia et al., 2020; Lin et al., 2021; Luo et al., 2021; Wang et al., 2021; Liao et al., 2022). Thus, autophagy plays important roles in plant growth and development and stress responses to facilitate plant tolerance and survival under unfavorable conditions (Marshall and Vierstra, 2018). In plants, autophagy is maintained at a basal level during growth and development to ensure homeostasis, but it is upregulated under environmental stresses to aid plant survival (Wang et al., 2018). The autophagy process starts with induction by upstream kinases, following by cargo recognition, phagophore formation, phagophore expansion and closure, autophagosome fusion and breakdown in vacuoles (Marshall and Vierstra, 2018).

The core machinery of autophagy is conserved among eukaryotes and is mainly regulated by two energy sensors, Sucrose Non-Fermenting (SNF)-related kinase 1 (SnRK1) and Target of Rapamycin (TOR). In Arabidopsis, SnRK1 is required for autophagy induction under a wide variety of stress conditions (Soto-Burgos and Bassham, 2017). SnRK1 activates autophagy through direct phosphorylation of ATG1 and through inhibition of TOR activity, and functions upstream of TOR (Chen et al., 2017; Soto-Burgos and Bassham, 2017). In contrast, TOR kinase functions as a negative regulator of plant autophagy (Liu and Bassham, 2010). In the case of TOR inactivation, the transcripts of many autophagy-related genes (*ATGs*) were significantly up-regulated in Arabidopsis, leading to the activation of autophagy machinery (Liu et al., 2012; Dong et al., 2015). In yeast and Arabidopsis, TOR regulates autophagy through direct phosphorylation of ATG13 (Alers et al., 2012; Son et al., 2018; van Leene et al., 2019).

FERONIA and TOR have both been shown to positively regulate plant growth. Very recently, the crosstalk between FER-mediated signaling and TOR-mediated nutrient/energy metabolism has been reported, which revealed that FER interacts with the TOR pathway to regulate nitrogen-related nutrient signaling under low nutrient conditions (Song et al., 2022). However, how FER regulates autophagy and whether or not this regulation is through TOR is unknown. In this study, we investigated autophagy phenotype of *fer-4* mutant, and our genetic studies showed strong interaction between FER and TOR in plant growth and autophagy. We further found that FER is required for TOR kinase activity. Our results showed that FER functions through TOR to negatively regulate autophagy.

## Materials and methods

### Plant materials and growth conditions

The Arabidopsis accession Columbia-0 was used as WT in all experiments. T-DNA insertion mutants *fer-4* (GABI-106A06), *TOROE* (GABI_548G07), *raptor1b* (SALK_078159), s*6k1* (SALK_148694) were described previously (Anderson et al., 2005; Deprost et al., 2007; Henriques et al., 2010; Ren et al., 2011; Guo et al., 2018). *GFP-ATG8e* overexpression line was previously described (Xiong et al., 2007). For all experiments involving Arabidopsis plants, seeds were sterilized with 70% ethanol containing 0.1% Triton and germinated on 1/2 Linsmainer and Skoog (LS) plates with 1% sucrose and 0.7% agar, with or without treatments as indicated when it is appropriate. 10-day old seedlings were transferred into soil at 22°C under long-day (16 h light/8 h dark) conditions with a photon fluence rate of ∼120 μmol m^–2^s^–1^.

### Sucrose starvation and autophagosome observation

For autophagosome observation and quantification, 7-day-old seedlings grown under constant light were transferred to control 1/2 LS plates with 1% sucrose and starvation plates without sucrose, and incubate in dark for three days. GFP-ATG8e-labeled autophagosomes were observed and counted by epifluorescence microscopy using a fluorescein isothiocyanate (FITC) filter. Representative GFP-labeled autophagosome images were taken by confocal microscopy using a Zeiss Laser Scanning Microscope 700 (LSM700) with a 63 × oil immersion objective. GFP was excited with a 488 nm laser line and detected from 555 nm.

### AZD8055 treatment

For root growth inhibition assay, sterilized seeds were germinated on control 1/2 LS or plates containing 1 μM AZD8055 for 7 days under constant light. The plates were scanned and the root lengths were measured using ImageJ.

For short term AZD8055 treatment for western blotting, 10-day-old seedlings grown on 1/2 LS plates were transferred to control liquid 1/2 LS or 1/2 LS with AZD8055 for the amount of time indicated in each experiment. The seedlings were collected and dabbed dry and flash frozen in liquid nitrogen. The samples were ground in 2xSDS buffer (100 mM Tris–HCl pH 6.8, 4% (w/v) SDS, 20% (v/v) glycerol, 0.2% (w/v) bromophenol blue, 0.2 M β-mercaptoethanol) and used for immunoblot analysis using anti-S6K1/2 antibody (Agrisera, AS121855). The relative intensity of P-S6K and S6K were quantified using ImageJ.

For short term AZD8055 treatment for autophagosome observation, 7-day-old seedlings of *WT GFP-ATG8e* and *fer-4 GFP-ATG8e* grown on 1/2 LS plates were transferred to control liquid 1/2 LS or 1/2 LS with 1 μM AZD8055 for 6 hours before observation under an epifluorescence microscope.

### GFP-ATG8e cleavage assay

Seven-day-old *WT GFP-ATG8e* and *fer-4 GFP-ATG8e* seedlings were subjected to 1/2 LS liquid medium without sucrose for 6, 12, and 24 hours in dark, or to 1/2 LS liquid with or without 1 μM AZD8055 for 6 hours in light before harvesting. Total protein was isolated with 2xSDS buffer (100 mM Tris–HCl pH 6.8, 4% (w/v) SDS, 20% (v/v) glycerol, 0.2% (w/v) bromophenol blue, 0.2 M β-mercaptoethanol) and resolved on 10% SDS-PAGE gels for immunoblot analysis using monoclonal anti-GFP antibody (Sigma-Aldrich, MAB3836). The relative intensity of GFP-ATG8e and free GFP were quantified using ImageJ.

### Protoplast isolation and transient expression assays

Leaves of 4-week-old Col-0 and *raptor1b* plants grown under long-day conditions were collected and peeled for protoplast isolation as previously described (Yoo et al., 2007; Wu et al., 2009). Protoplasts were resuspended in MMg solution (400 mM mannitol, 15 mM MgCl_2_, and 4 mM MES pH5.7). Plasmids of *35S:GFP* control, *35S:FER-GFP* and *35S:mCherry-ATG8e* were prepared using Maxiprep kits (Sigma-Aldrich, NA0310) and set the final concentration at 1 μg/μL. Ten micrograms of each plasmid was introduced into 200 μL protoplasts by adding 220 μL of PEG solution (40% PEG4000, 200 mM mannitol, and 100 mM CaCl_2_). After transformation, protoplasts were washed and incubated in 1 mL of W5 solution (154 mM NaCl, 125 mM CaCl_2_, 5 mM KCl, 2 mM MES pH5.7) overnight.

For sucrose starvation, protoplasts were incubated in W5 solution without sucrose or with 0.5% (w/v) sucrose as control for 36 hours at room temperature. For constitutive autophagy in *raptor1b* mutant, protoplasts were incubated in W5 solution only for 20 hours. Protoplasts were observed using an epifluorescence microscope (Carl Zeiss Axio Imager.A2, Germany) with TRITC filter. Protoplasts with more than three visible autophagosomes were counted as being active for autophagy. A total of 100 well-expressed protoplasts were observed per treatment per genotype, and the percentage of protoplasts with active autophagy was quantified and averaged from 3-4 independent replicates (Wang et al., 2021).

### Generalized linear model

To specifically examine the genotype-by-treatment interaction to determine the sensitivity of the *fer-4*, TOROE, and *fer-4 TOROE* to AZD treatment, we used a generalized linear model (glm package in R) with genotype and treatment (Control or AZD) as fixed factors. Genotypes were categorized as significantly differentially responsive to AZD if they had a genotype-by-treatment interaction *p*-value < 0.05 compared to all other lines.

### Statistical analysis

Graphs were created in GraphPad Prism software (version 9.3.0). SPSS version 27.0 software (IBM, Armonk, NY, United States) was used for statistical data analysis. The data are shown as means ± standard error of the mean (SEM) and were subjected to one-way analysis of variance (ANOVA) Tukey’s multiple range tests (*p* < 0.05).

## Results

### FER negatively regulates autophagy

**FIGURE 1:**
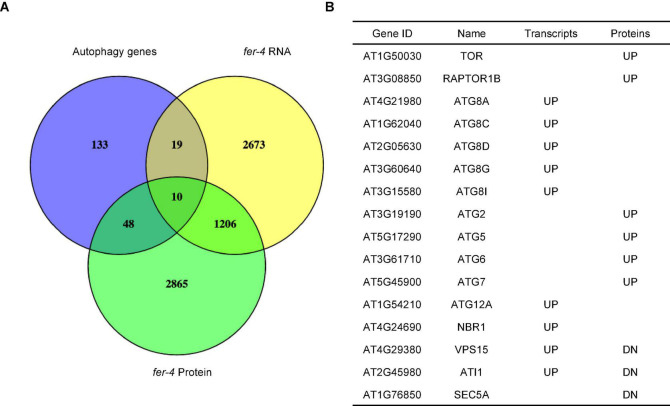
Comparison of autophagy-related genes with transcriptome and proteome of *fer-4*. **(A)** Venn diagram showing the overlaps among autophagy-related genes (Homma et al., 2011) and differentially expressed transcripts and proteins in *fer-4* mutant. **(B)** Selected genes involved in autophagy and their changes in two sets of differentially expressed data (UP: increased levels in *fer-4* mutant; DN: decreased levels in *fer-4* mutant).

Our recently published multi-omics analysis of the loss-of-function *fer-4* mutant revealed that the Gene Ontology term, autophagy (GO:0006914) is enriched (Wang et al., 2022), suggesting that FER plays a role in autophagy regulation. We then compared *fer-4* transcriptome and proteome to autophagy-related genes (Homma et al., 2011). The analysis revealed that about 36% (77/212) of the genes known to be involved in plant autophagy have altered levels of transcripts or proteins in *fer-4* mutant (Figure 1A; Supplementary Table 1). Closer examination showed that at least 16 genes with known functions in autophagy have altered transcripts or protein levels in *fer-4*, including many autophagy-related genes (*ATGs*), Target of Rapamycin (TOR) and Regulatory-associated protein of TOR 1B (RAPTOR1B) (Figure 1B).

To gain a better understanding of the role FER plays in autophagy, we crossed *GFP-ATG8e* transgenic plants with *fer-4* mutant to generate *fer-4 GFP-ATG8e*. ATG8e is localized on the autophagic membranes and can serve as a reliable marker for autophagosome observation and quantification (Contento et al., 2005). We carried out sucrose starvation followed by autophagosome quantification in 10-day-old Arabidopsis seedlings.

**FIGURE 2:**
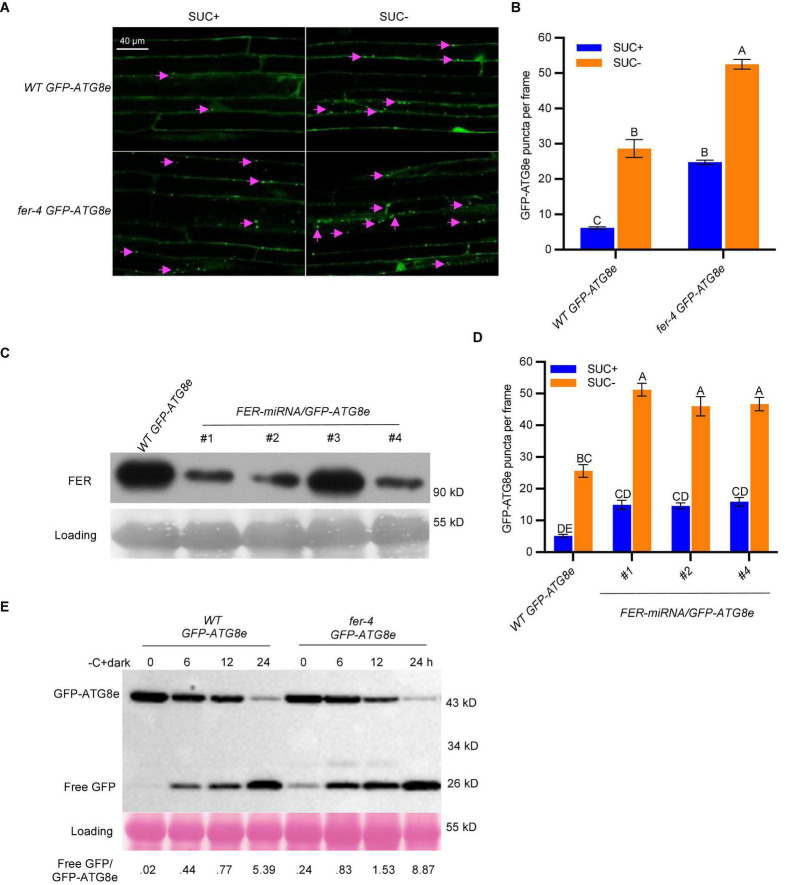
FER negatively regulates autophagy under sucrose starvation. **(A)** Representative confocal microscopic images of WT and *fer-4* with autophagosome marker GFP-ATG8e, under control (SUC +) and sucrose starvation (SUC-) conditions. The Lavender arrows indicate autophagosomes. Scale = 40 μm. **(B)** Quantification of autophagosomes of the 10-day-old seedlings from **(A)**. Data are shown as means ± SEM from 3 biological replicates, with 6-12 images per replicate. Different letters indicate significant difference among groups subjected to one-way ANOVA Tukey’s multiple range tests (*p* < 0.05). **(C)** Western blot showing the decreased FER protein levels in multiple T2 lines of *FER-miRNA/GFP-ATG8e* using anti-FER antibody (Guo et al., 2018). Lines #1, #2 and #4 were used for further autophagy analysis. Rubisco was used as loading control. **(D)** Quantification of autophagosomes of the 10-day-old seedlings from C with or without sucrose and grown in dark for 3 days. Data are shown as means ± SEM from 5-12 seedlings, with 2-3 images per seedlings. Different letters indicate significant difference among groups subjected to one-way ANOVA Tukey’s multiple range tests (*p* < 0.05). **(E)** GFP-ATG8e cleavage assays showing the increased autophagic flux in *fer-4* mutant. Seven-day-old *WT GFP-ATG8e* and *fer-4 GFP-ATG8e* seedlings were subjected to 1/2 LS liquid medium without sucrose for 6, 12, and 24 hours in dark. The total proteins were extracted and separated by SDS-PAGE gels followed by immunoblotting with anti-GFP antibody. Ponceau S staining serves as protein loading control. The values below the blots are the ratios of free GFP over GFP-ATG8e. This experiment was carried out two times with similar results.

While very few autophagosomes were evident in *WT GFP-ATG8e* under normal growth conditions, the number was substantially increased by sucrose starvation. Interestingly, the *fer-4 GFP-ATG8e* displayed constitutive autophagy, with significantly increased levels of autophagosomes under both normal and starvation conditions (Figures 2A,B). The results indicated that FER functions to suppress autophagy pathway. To further support that FER functions to inhibit autophagy pathway, we generated a second FER mutant allele, *FER-miRNA/GFP-ATG8e*, by overexpressing *FER-miRNA* in *WT GFP-ATG8e* plants. *FER-miRNA* is highly effective in knocking down the endogenous FER (Guo et al., 2009; Wang et al., 2022). Three T2 transgenic lines (*FER-miRNA/GFP-ATG8e #1, #2, #4*), in which the FER protein levels were significantly decreased, were used for sucrose starvation followed by autophagosome quantification (Figure 2C). Similar to *fer-4 GFP-ATG8e*, all three lines had higher levels of autophagosomes than *WT GFP-ATG8e* under both nutrient-rich (SUC +) and sucrose starvation (SUC-) conditions (Figure 2D).

Upon autophagosome fusion with vacuole, the GFP-ATG8e protein is delivered to vacuole where it is degraded to release a free and relatively stable GFP; and the free GFP/GFP-ATG8e ratio reflects the level of autophagy activity (Li et al., 2014; Chen et al., 2017). Seven-day-old *WT GFP-ATG8e* and *fer-4 GFP-ATG8e* seedlings were treated in sucrose-free 1/2 LS medium in dark for 0, 6, 12, and 24 h. As shown in Figure 2E, the release of free GFP was readily observed in both WT and *fer-4* mutant plants upon carbon starvation, whereas the free GFP/GFP-ATG8e ratios in the *fer-4* mutant were higher than that in WT, even at 0 hour control condition, suggesting that there was constitutive autophagy in *fer-4* mutant and depletion of FER enhances autophagic activity. These results demonstrated that FER negatively regulates autophagy.

**FIGURE 3:**
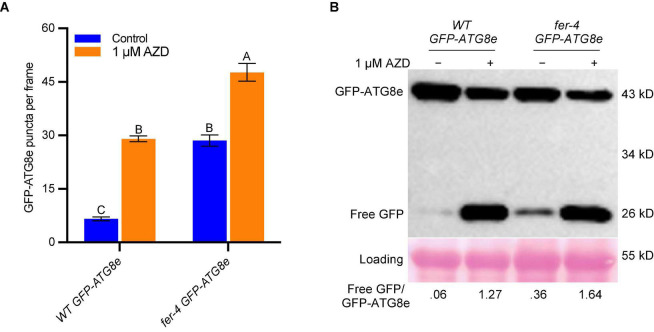
FER negatively regulates autophagy under AZD8055 treatment. **(A)** Quantification of autophagosomes. Seven-day-old seedlings of *WT GFP-ATG8e* and *fer-4 GFP-ATG8e* grown on 1/2 LS plates were transferred to control liquid 1/2 LS or 1/2 LS with 1 μM AZD8055 for 6 h. Data are shown as means ± SEM from 11-13 seedlings, with 2-3 images per seedlings. Different letters indicate significant difference among groups subjected to one-way ANOVA Tukey’s multiple range tests (*p* < 0.05). **(B)** GFP-ATG8e cleavage assays of seedlings from A. The total proteins were extracted and separated by SDS-PAGE gels followed by immunoblotting with anti-GFP antibody. Ponceau S staining serves as protein loading control. The values below the blots are the ratios of free GFP over GFP-ATG8e. This experiment was carried out two times with similar results.

TOR is a negative regulator of autophagy in plants (Liu and Bassham, 2010) and its kinase inhibitor AZD8055 was used to induce autophagy by suppressing TOR activity (Dong et al., 2015; Pu et al., 2017). We also applied 1 μM AZD8055 to 7-day-old *WT GFP-ATG8e* and *fer-4 GFP-ATG8e* seedlings for 6 hours for autophagosomes observation and GFP-ATG8e cleavage assay. Similar to sucrose starvation, the *fer-4 GFP-ATG8e* had significantly more autophagosomes (Figure 3A) and higher free GFP/GFP-ATG8e ratios (Figure 3B) than *WT GFP-ATG8e* under both control and AZD8055 treatment conditions. These results further supports that FER negatively regulates autophagy.

### FER is required for TOR kinase activity

TOR is an atypical Ser/Thr kinase of the phosphatidylinositol 3-kinase-related lipid kinase family. Similar to its counterparts in yeast and mammals, TOR functions in a complex with RAPTOR and LST8 and plays central roles in balancing nutrient, energy, and internal and external stimuli to regulate plant growth development and stress responses (Shi et al., 2018). The fact that both TOR and FER positively regulate plant growth and negatively regulate autophagy (Figures 2–3; Deprost et al., 2007; Guo et al., 2009; Liu and Bassham, 2010; Pu et al., 2017; Soto-Burgos and Bassham, 2017) prompted us to examine their interactions more closely.

**FIGURE 4:**
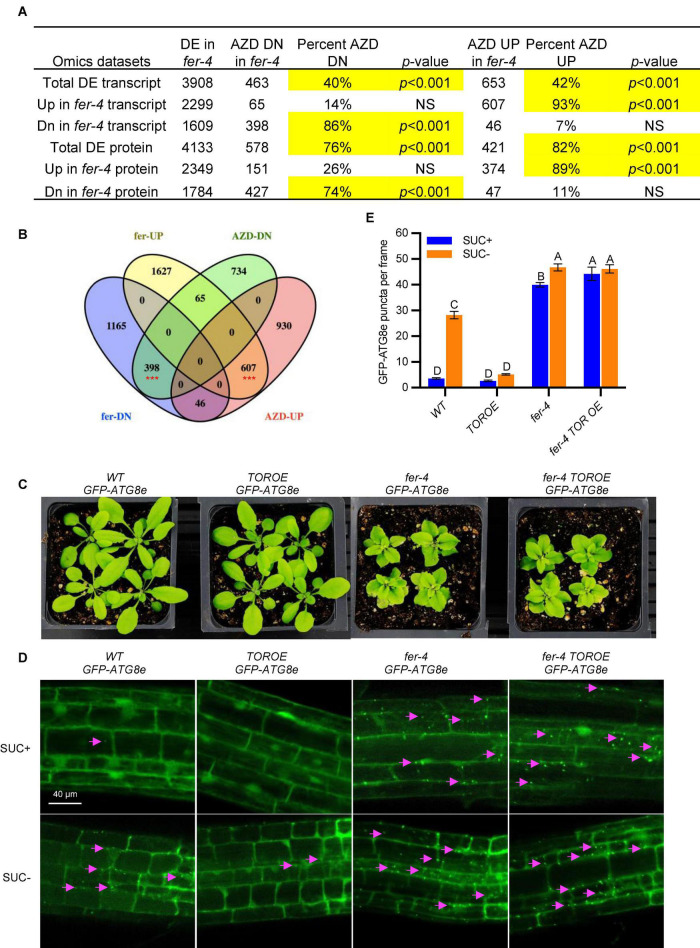
FER and TOR cooperate in plant growth and autophagy. **(A)** Hypergeometric tests on the comparisons of AZD-regulated transcripts and differentially expressed transcripts or proteins in *fer-4* mutant. Significant overlaps are highlighted in yellow. DE: differentially expressed; NS: not significant. **(B)** Venn diagram showing the comparison of differentially expressed transcripts in *fer-4* and differentially expressed genes in response to TOR kinase inhibitor AZD8055 treatment (Dong et al., 2015). The red *** represents the overlaps statistically significant with *p* < 0.001. **(C)** Growth phenotype of 3-week-old plants of WT, *fer-4*, *TOROE* and *fer-4 TOROE* double mutant. **(D)** Representative confocal microscopic images of WT, *fer-4*, *TOROE* and *fer-4 TOROE* with autophagosome marker GFP-ATG8e, under control (SUC +) and sucrose starvation (SUC-) conditions. The Lavender arrows indicate the autophagosomes. Scale = 40 μm. **(E)** Quantification of autophagosomes in different genotypes expressing GFP-ATG8e from 10-day-old seedlings with or without sucrose for 3 days. Data are shown as means ± SEM from 3 biological replicates, with 14-31 images per replicate. Different letters indicate significant difference among groups subjected to one-way ANOVA Tukey’s multiple range tests (*p* < 0.05).

The comparison of genes mis-expressed in *fer-4* (Wang et al., 2022) and the genes regulated by AZD8055 (Dong et al., 2015) revealed that a large number of TOR-dependent genes (40% of AZD-repressed, *p* < 0.001; 42% of AZD-induced, *p* < 0.001) are regulated by FER (Figures 4A,B). Interestingly, majority of the 40% of AZD-repressed genes (86%, *p* < 0.001) have decreased transcript levels in *fer-4*, and majority of the 42% of AZD-induced genes (93%, *p* < 0.001) have increased transcript levels in *fer-4*, which suggests a corporative relationship between FER and TOR (Figures 4A,B and Supplementary Tables 2–3). Comparisons with differentially expressed proteins in *fer-4* gave similar results (Figure 4A and Supplementary Tables 2, 3). We also compared AZD8055 regulated genes with two other sets of published transcriptome data of *fer-4*, one was from 10-day-old seedlings in Wang et al. (2022) and another was in Guo et al. (2018). The amount of overlaps are very similar, which further strengthened our observation that FER and TOR interact in a cooperative manner (Supplementary Figure 1).

We further constructed the double mutant *fer-4 TOROE GFP-ATG8e*, where *TOROE* is a TOR overexpression T-DNA insertional mutant (Deprost et al., 2007). While the *TOROE* mutant growth phenotype is similar to that of WT and has decreased autophagy induction by sucrose starvation, *fer-4 TOROE GFP-ATG8e* mirrors *fer-4 GFP-ATG8e*, with stunted growth and constitutive autophagy (Figures 4C–E), which suggests that either FER is epistatic to TOR or TOR activity requires FER.

**FIGURE 5:**
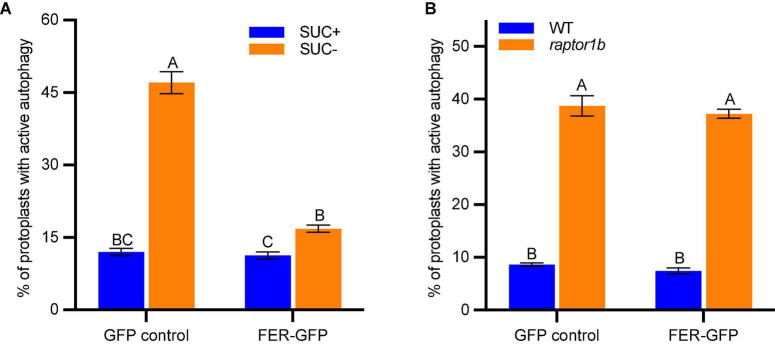
*FER-GFP* inhibits sucrose starvation-induced autophagy but not the constitutive autophagy in *raptor1b*. **(A,B)** Quantification of protoplasts with ATG8e labeled autophagy. **(A)** GFP control or *FER-GFP* was co-expressed with *mCherry-ATG8e* in Col-0 protoplasts, treated without or with 0.5% (w/v) sucrose for 36 h before microscopy. B, GFP control or *FER-GFP* was co-expressed with *mCherry-ATG8e* in Col-0 and *raptor1b* protoplasts, followed by 20 h incubation before microscopy. Protoplasts with more than three visible autophagosomes were counted as being active for autophagy. A total of 100 well-expressed protoplasts were observed for each treatment per genotype, and the percentage of protoplasts with active autophagy was calculated. Data are shown as means ± SEM from 3-4 biological replicates. Different letters indicate significant difference among groups subjected to one-way ANOVA Tukey’s multiple range tests (*p* < 0.05).

To clarify the genetic relationship between FER and TOR, we carried out a transient assay by co-expressing *FER-GFP* and autophagy marker *mCherry-ATG8e* in protoplasts. We first found that *FER-GFP* transient expression in Col-0 protoplasts could dramatically inhibit autophagy induction by sucrose starvation (Figure 5A). There was constitutive autophagy in *raptor1b* protoplasts even without any treatment compared with WT (Figure 5B; Pu et al., 2017). However, *FER-GFP* overexpression could not inhibit such constitutive autophagy in *raptor1b* (Figure 5B), suggesting that RAPTOR1B functions downstream of FER. This result suggests that TOR/RAPTOR1B complex functions downstream of FER, and the previous observation in the *fer TOROE* double mutant is likely due to that FER is required for TOR activity.

**FIGURE 6:**
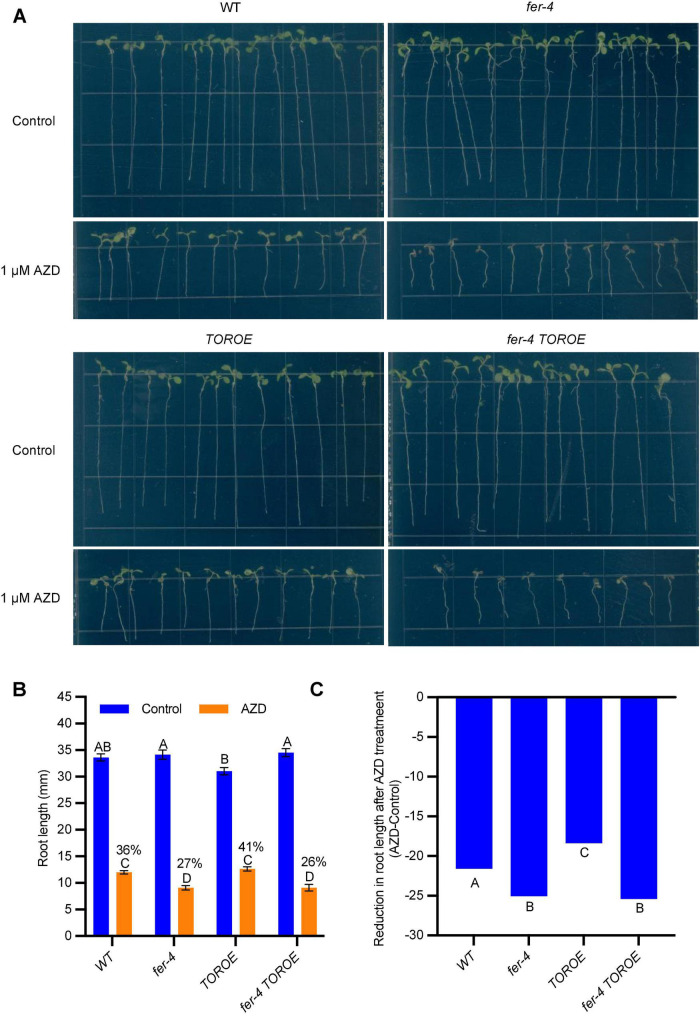
*fer-4* was more sensitive to AZD in root growth assay. **(A)** Images of 7-day-old seedlings of WT, *fer-4*, *TOROE* and *fer-4 TOROE* grown on control 1/2 MS plates or plates containing 1 μM AZD8055. **(B)** Root length measured from seedlings of A using ImageJ. Data are shown as means ± SEM (*n* = 12). Different letters indicate significant difference among groups subjected to one-way ANOVA Tukey’s multiple range tests (*p* < 0.05). **(C)** Root growth inhibition by AZD8055 was analyzed using a generalized linear model (glm package in R) with genotype and treatment (Control or AZD) as fixed factors. The genotypes were categorized as significantly differentially responsive to AZD if they had a genotype-by-treatment interaction *p*-value < 0.05 compared to all other genotypes (*n* = 12).

To further clarify that TOR requires FER for its activity, we carried out a root growth inhibition assay in response to TOR kinase inhibitor AZD8055. After growing on control 1/2 LS plates or plates containing 1 μM AZD8055 for 7 days, WT root growth was inhibited by 1 μM AZD8055 to 36% of the control treatment, while *fer-4* was hypersensitive to the inhibitor treatment, with root growth decreased to 27% of the control, suggesting decreased TOR activity in *fer-4* mutant. As expected, *TOROE* is less sensitive to AZD8055 than that of WT with 41% growth, and *fer-4 TOROE* double mutant is more sensitive to the inhibitor, with 26% growth of the control, similar to *fer-4* mutant (Figures 6A,B).

To ascertain that *fer-4 TOROE* responded in a similar fashion to *fer-4* single mutant in AZD8055-mediated root inhibition, we also analyzed the responses of WT, *fer-4*, *TOROE*, and *fer-4 TOROE* to AZD8055 using a generalized linear model (glm package in R) with genotype and treatment (Control or AZD) as fixed factors. Similar results were obtained that *fer-4* and *fer-4 TOROE* are more sensitive to AZD8055 treatment while *TOROE* is more resistant to the treatment significantly (Figure 6C).

**FIGURE 7:**
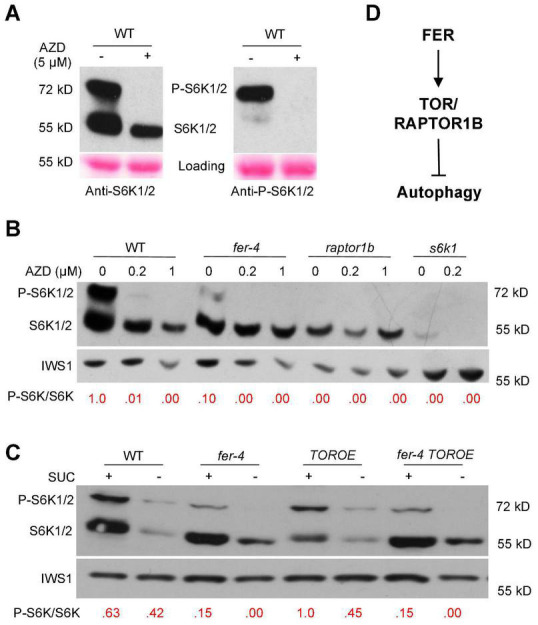
S6K1/2 phosphorylation by TOR was decreased in *fer-4* mutant. **(A)** Western blot showing testing of anti-S6K1/2 and anti-P-S6K1/2 (phosphorylated S6K1/2) antibodies in WT with or without 5 μM AZD8055 for 3 h. Ponceau S staining was used as loading control. **(B,C)** Western blot showing phosphorylated and non-phosphorylated S6K1 in 10-day-old seedlings of indicated genotypes, with and without different concentrations of AZD8055 treatment for 1 h **(B)** or under control or sucrose-starvation conditions **(C)**. The ratios of P-S6K1/S6K1 were obtained using ImageJ, and the ratio in WT was set as 1.0 in **(B)** and 1.0 for *TOROE* in **(C)**. IWS1 protein was used as loading control with anti-IWS1 (Wang et al., 2021). **(D)** A working model showing a possible mechanism in which FER activates TOR/RAPTOR1B to inhibit autophagy.

S6K1/2, P70 ribosomal S6 kinases, are substrates of TOR kinase and their phosphorylation status can be used as an indicator of TOR kinase activity (Mahfouz et al., 2006). To further examine TOR activity in the *fer-4* mutant, we obtained anti-S6K1/2 and anti-P-S6K1/2 antibodies from Agrisera and tested in WT with and without AZD8055 treatment. While anti-P-S6K1/2 antibody only recognized the phosphorylated S6K1/2, anti-S6K1/2 antibody recognized both phosphorylated and non-phosphorylated S6K1/2 (Figure 7A). AZD8055 (5 μM) treatment for three hours completely diminished the phosphorylated S6K1/2 (Figure 7A), confirming that S6K phosphorylation can be used as a readout of TOR activity. The anti-S6K1/2 antibody can recognize both forms of S6K1 and has high specificity since very little signal (likely from S6K2) was observed in *s6k1* mutant (Figure 7B), and it was therefore used for subsequent experiments.

Western blotting was carried out using 10-day-old seedlings with and without treatment of different AZD8055 concentrations for one hour. Compared to WT, *fer-4* mutant has decreased TOR kinase activity, with lowered P-S6K1/S6K1 ratio. The *raptor1b* mutant, where TOR activity is severely compromised, has no detectable phosphorylated S6K1 (Figure 7B). The TOR OE plants have relatively higher P-S6K1/S6K1 ratio than that in WT after AZD8055 treatment (Supplementary Figure 2). We further examined the TOR kinase activity in *fer-4 TOROE* double mutant. Under normal growth conditions supplemented with sucrose, while *fer-4* has decreased and *TOROE* has elevated TOR kinase activity, the double mutant behaves like *fer-4*, with decreased TOR kinase activity reflected by lowered P-S6K1/S6K1 ratio, compared to WT control. Sucrose starvation for three days decreased both forms of the S6K1 protein level (Figure 7C). Taken together, these results support the hypothesis that FER is required for TOR kinase activity and its normal function.

In summary, our results demonstrated that FER is required for TOR kinase activity, and FER functions through TOR kinase to negatively regulate autophagy (Figure 7D). Thus, our results provide novel insights into the regulation of autophagy by a plasma membrane-localized receptor-like kinase FER.

## Discussion

FERONIA receptor kinase is a versatile regulator and mediates many important biological processes in plant growth, development and stress responses. Our recent integrated omics analysis of *fer-4* mutant not only confirmed the previous known FER functions but also revealed novel pathways regulated by FER and underlying molecular mechanisms, such as ER body formation, indole glucosinolate biosynthesis and ABA responses (Wang et al., 2022). In this study, we further expanded the findings and provided genetic, molecular and cell biological evidence that FER is involved in autophagy regulation. Consistent with the significant overlaps between autophagy genes and those affected in *fer-4* mutant (Figure 1), *fer* mutants displayed constitutive autophagy phenotype under normal condition, sucrose starvation and AZD8055 treatments (Figures 2, 3). Further genetic, cell biology and biochemical assays showed that FER is required for TOR kinase activity (Figures 4–7). Our results indicated that FER functions through TOR to negatively regulate autophagy.

As a conserved cellular process, autophagy plays important roles in recycling cytosolic material and maintaining cellular homeostasis during growth, development and responses to diverse environmental stresses. TOR, as an essential serine/threonine kinase, is a master regulator of autophagy. From cargo selection to the destination vacuole, autophagy involves many steps, and TOR plays important roles in integrating the nutrient and energy signals to initiate autophagy (Liu and Bassham, 2012; Marshall and Vierstra, 2018; Soto-Burgos et al., 2018). Despite all of the progress, how TOR is regulated by signaling pathways is less known. This study provides new insights into how TOR is regulated by a plasma membrane-localized receptor kinase, capable of integrating internal and external stimuli, to regulate autophagy.

FERONIA has been shown to physically interact with TOR kinase complex (Martínez Pacheco et al., 2022; Song et al., 2022). It is conceivable that FER and TOR also directly interact during autophagy regulation. Another possible mechanism by which FER regulates TOR kinase activity is through a Rho GTPase, ROP2. ROP2 has been shown to directly interact and activate TOR in response to auxin and light (Li et al., 2017; Schepetilnikov et al., 2017). FER was shown to directly interact with ROP2 and activate ROP2-mediated signaling (Duan et al., 2010). It is also conceivable that FER functions in the same complex with ROP2 and TOR, and thus activates TOR kinase.

We previously observed that TOR and RAPTOR1B protein levels were increased in *fer-4* mutant (Figure 1B) while TOR has reduced activity in the mutant (Figures 6, 7). This seemingly conflicting observation could be due to the possibility that kinase activity is negatively correlated to its protein stability, lower TOR kinase activity stabilizes the protein. This is also observed in the case of FERONIA kinase. When transiently expressed in *Nicotiana Benthamiana*, FER^*K*565*R*^, an inactive kinase accumulated to much higher levels compared to the wild-type FER (Guo et al., 2018; Wang et al., 2022). When transformed to *fer-4* for complementation, FER^*K*565*R*^ protein levels are in general higher that wild-type FER, whereas FER^*K*565*R*^ could not complement the mutant to the same extent as the wild-type FER (Chakravorty et al., 2018).

Our multi-omics data analysis with *fer-4* mutant produced many enriched GO terms related to nutrient and energy production and metabolism (Wang et al., 2022), suggesting that FER plays important roles in nutrient and energy homeostasis. Our study therefore suggests an exciting possibility that FER and TOR function together to gauge nutrient and energy levels and regulate autophagy induction. Consistent with this possibility, a recent study showed that RALF1-FER complex interacts with and activates TOR signaling in response to nitrogen starvation (Song et al., 2022). FER regulates diverse biological processes, and autophagy is a critical cellular recycling process involved in plant growth development and stress responses. We show here that FER is required for TOR kinase activity and FER functions through TOR to regulate autophagy. How FER/TOR-regulated autophagy is involved in FER-regulated biological processes will be interesting for future studies.

## Data availability statement

The original raw data presented in this study are included in the article/Supplementary material, further inquiries can be directed to the corresponding author. The raw RNA and protein sequencing data are from our previous published article (Wang et al., 2022), which deposited the data at the Gene Expression Omnibus under series GSE143634 and GSE191303, and on MassIVE under series MSV000084804.

## Author contributions

HG conceived the project. HG and PW performed the genetic, molecular, biochemical, and cell biology analysis. GS performed the proteomics. NC, GS, and TN performed the omics data analysis. NC performed the statistical analysis for transcript and protein enrichment. OW, DB, and C-YL contributed to data analysis. HG and PW wrote the manuscript, with edits or input from other co-authors. All authors contributed to the article and approved the submitted version.

## Supplementary material

Supplementary Figure 1 Comparison of AZD8055 regulated genes with another two sets of published transcriptomes of *fer*-4. **(A)** Venn diagram comparison of differentially expressed transcripts in 10-day-old *fer*-4 seedlings (Wang et al., 2022) with differentially expressed genes in response to TOR kinase inhibitor AZD8055 treatment (Dong et al., 2015). **(B–C)** Venn diagram comparisons of the co-regulated genes from A and from Figure 4B. **(D)** Venn diagram comparison of differentially expressed transcripts in *fer*-4 published by Guo et al. (2018) with differentially expressed genes in response to TOR kinase inhibitor AZD8055 treatment (Dong et al., 2015). **(E–F)** Venn diagram comparisons of the co-regulated genes from **(D)** and from Figure 4B.

Supplementary Figure 2 S6K1 phosphorylation in WT and *TOR OE* seedlings. Seven-day-old seedlings were treated with 0.2 μM AZD8055 for 2 hours. Ponceau S staining was used as loading control. The ratios of P-S6K1/S6K1 were obtained using ImageJ, and the ratio in WT was set as 1.0.
