# Supplementary figures and images for: FERONIA functions through Target of Rapamycin (TOR) to negatively regulate autophagy
Source: Front Plant Sci. 2022 Aug 23;13:961096. doi: 10.3389/fpls.2022.961096 (PMC9446147; doi:10.3389/fpls.2022.961096)

**AZD DN**

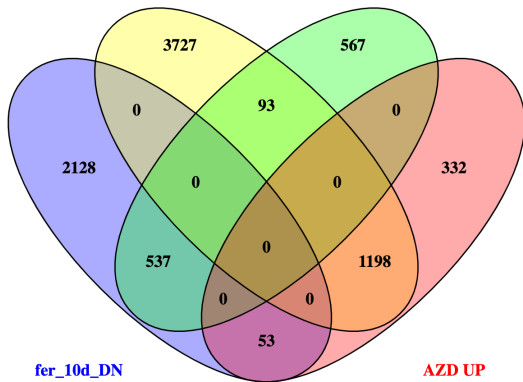

**Co\_UP\_1198**

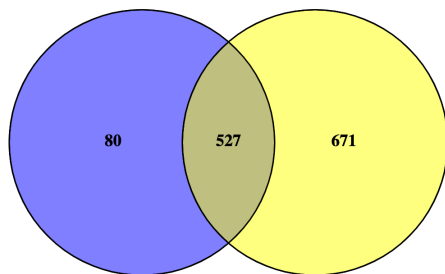

Co\_DN\_537

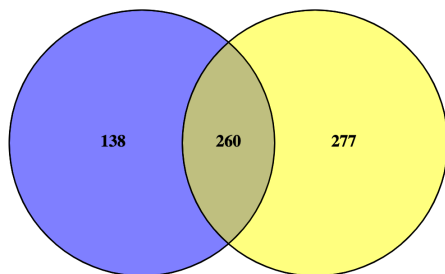**AZD DN**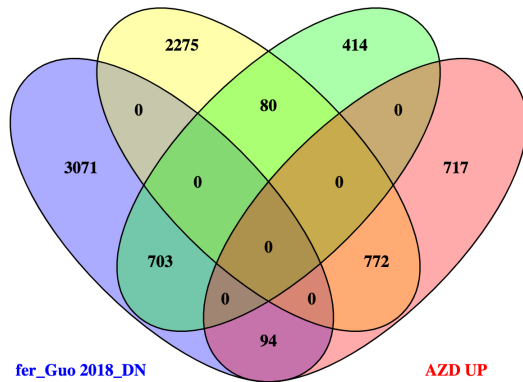

**Co\_UP\_772**

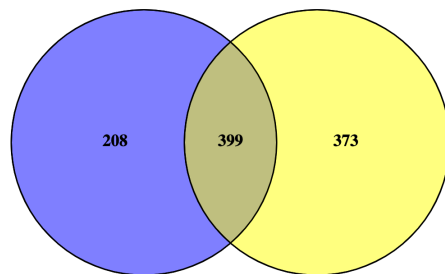

**Co\_DN\_703**

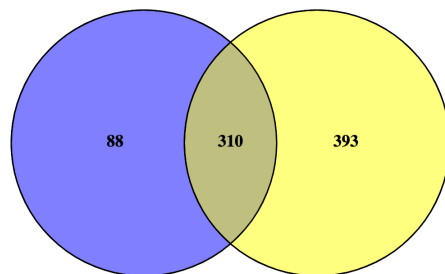

Supplement: Supplementary Figure 1 — Comparison of AZD8055 regulated genes with another two sets of published transcriptomes of fer-4. (A) Venn diagram comparison of differentially expressed transcripts in 10-day-old fer-4 seedlings (Wang et al., 2022) with differentially expressed genes in response to TOR kinase inhibitor AZD8055 treatment (Dong et al., 2015). (B–C) Venn diagram comparisons of the co-regulated genes from A and from Figure 4B. (D) Venn diagram comparison of differentially expressed transcripts in fer-4 published by Guo et al. (2018) with differentially expressed genes in response to TOR kinase inhibitor AZD8055 treatment (Dong et al., 2015). (E–F) Venn diagram comparisons of the co-regulated genes from (D) and from Figure 4B. [file Image_1.pdf]

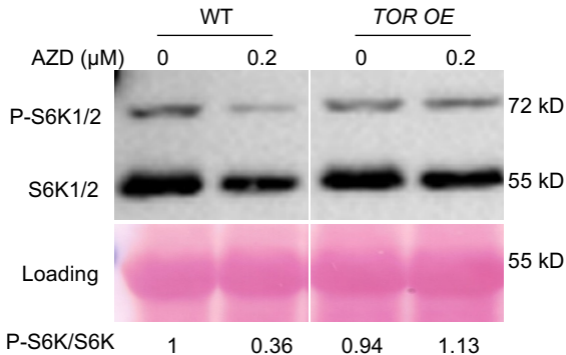

Supplement: Supplementary Figure 2 — S6K1 phosphorylation in WT and TOR OE seedlings. Seven-day-old seedlings were treated with 0.2 μM AZD8055 for 2 hours. Ponceau S staining was used as loading control. The ratios of P-S6K1/S6K1 were obtained using ImageJ, and the ratio in WT was set as 1.0. [file Image_2.pdf]
